# *Streptococcus mutans* Protein Synthesis during Mixed-Species Biofilm Development by High-Throughput Quantitative Proteomics

**DOI:** 10.1371/journal.pone.0045795

**Published:** 2012-09-25

**Authors:** Marlise I. Klein, Jin Xiao, Bingwen Lu, Claire M. Delahunty, John R. Yates, Hyun Koo

**Affiliations:** 1 Center for Oral Biology, University of Rochester Medical Center, Rochester, New York, United States of America; 2 Department of Microbiology and Immunology, University of Rochester Medical Center, Rochester, New York, United States of America; 3 State Key Laboratory of Oral Diseases, Sichuan University, Chengdu, People’s Republic of China; 4 The Scripps Research Institute, La Jolla, California, United States of America; Virginia Commonwealth University, United States of America

## Abstract

Biofilms formed on tooth surfaces are comprised of mixed microbiota enmeshed in an extracellular matrix. Oral biofilms are constantly exposed to environmental changes, which influence the microbial composition, matrix formation and expression of virulence. *Streptococcus mutans* and sucrose are key modulators associated with the evolution of virulent-cariogenic biofilms. In this study, we used a high-throughput quantitative proteomics approach to examine how *S. mutans* produces relevant proteins that facilitate its establishment and optimal survival during mixed-species biofilms development induced by sucrose. Biofilms of *S. mutans*, alone or mixed with *Actinomyces naeslundii* and *Streptococcus oralis*, were initially formed onto saliva-coated hydroxyapatite surface under carbohydrate-limiting condition. Sucrose (1%, w/v) was then introduced to cause environmental changes, and to induce biofilm accumulation. Multidimensional protein identification technology (MudPIT) approach detected up to 60% of proteins encoded by *S. mutans* within biofilms. Specific proteins associated with exopolysaccharide matrix assembly, metabolic and stress adaptation processes were highly abundant as the biofilm transit from earlier to later developmental stages following sucrose introduction. Our results indicate that *S. mutans* within a mixed-species biofilm community increases the expression of specific genes associated with glucan synthesis and remodeling (*gtfBC*, *dexA*) and glucan-binding (*gbpB*) during this transition (*P*<0.05). Furthermore, *S. mutans* up-regulates specific adaptation mechanisms to cope with acidic environments (F1F0-ATPase system, fatty acid biosynthesis, branched chain amino acids metabolism), and molecular chaperones (GroEL). Interestingly, the protein levels and gene expression are in general augmented when *S. mutans* form mixed-species biofilms (vs. single-species biofilms) demonstrating fundamental differences in the matrix assembly, survival and biofilm maintenance in the presence of other organisms. Our data provide insights about how *S. mutans* optimizes its metabolism and adapts/survives within the mixed-species community in response to a dynamically changing environment. This reflects the intricate physiological processes linked to expression of virulence by this bacterium within complex biofilms.

## Introduction

Virulent biofilms formed on surfaces are associated with many human infections, including those occurring in the mouth. The disease dental caries is a prime example of the consequences arising from complex interactions that occur in the mouth between specific oral bacteria (and their products), host saliva and dietary carbohydrates. These interactions modulate the transition from a healthy condition to a disease state (dental caries) by promoting the establishment of cariogenic biofilms on the susceptible tooth surface. The cariogenic biofilm consist of a highly acidogenic and aciduric microbiota enmeshed in an exopolysaccharides (EPS)-rich matrix, which creates a highly adhesive, cohesive and acidic milieu [1], [2], [3], [4]. The acidic milieu eventually leads to the clinical onset of cavitation through acid demineralization of the adjacent enamel.

Exopolysaccharides are key components of the matrix and proven virulence factors associated with pathogenesis of dental caries [5], [6]. In the mouth, a complex and diverse microbial community is interacting with the pellicle on tooth surfaces, to which a small group of organisms (oral *Streptococci* and *Actinomyces* spp) can adhere [7], [8], [9], [10]. Cariogenic organisms such as *S. mutans* can also be present in this initial colonizing community, albeit usually in low number [1], [7], [10]. However, environmental changes such as frequent consumption of dietary sucrose dramatically influence the development of cariogenic biofilms, by providing a substrate for EPS synthesis and acid production.

The EPS are formed *in situ* by glucosyltransferases (Gtfs) from *S. mutans* present on the tooth-pellicle and bound on bacterial surfaces [4], [10]. The surface-formed polymers provide bacterial binding sites for subsequent colonization and local accumulation of *S. mutans* and other organisms on the tooth surface [4]. If sucrose is constantly available, the continuous EPS production further enmeshes the microbial cells forming microcolonies, resulting in a highly structured three dimensional (3D) matrix in virulent-cariogenic biofilms [11], [12], [13]. In parallel, the bacterial cells embedded in the matrix and clustered within 3D microcolonies can convert sucrose (and other fermentable carbohydrates) into acids, which in turn are trapped within EPS-rich milieu [13]. The resulting low pH microenvironment selects aciduric organisms [1], [2]. Therefore, *S. mutans* and sucrose are key modulators for the evolution of cariogenic biofilms.

As the environmental pH, EPS content and microbial composition change in the complex 3D architecture, the molecular pathways required for optimal metabolism and survival of *S. mutans* may be adapted in response to a dynamically changing milieu allowing this bacterium to thrive. How these changes mediate *S. mutans* responses at protein level particularly in the context of ecological biofilm concept [1] and within a highly cohesive and acidic 3D environment [13] remain to be elucidated. Although much has been known about *S. mutans* physiology and gene expression, most research has been done in planktonic or single-species biofilms without regard to the complex microbial and biochemical changes occurring simultaneously. Therefore, we used a mixed-species ecological biofilm model [12], [13] and MudPIT, a powerful and innovative method [14], [15], to comprehensively characterize *S. mutans* proteome profile over the course of biofilm formation and maturation.

## Materials and Methods

### Bacterial Strains

*Streptococcus mutans* UA159, serotype c (ATCC 700610), *Actinomyces naeslundii* ATCC 12104 and *Streptococcus oralis* ATCC 35037 were used for biofilm formation. These representative organisms found in the mouth have been previously used to mimic the ecological biofilm concept [12], [13] (Figure S1). *Streptococcus oralis* and *Actinomyces naeslundii* are well established early colonizers in the mouth while *S. mutans* is a proven virulent (cariogenic) oral pathogen. Furthermore, *Streptococcus oralis* ATCC 35037 produces soluble glucans, and can be highly acid tolerant [16]. *A. naeslundii* may be associated with development of root caries [17]; the strain 12104 is acidogenic and produces EPS (i.e. fructans) [18], [19]. The cultures were stored at –80°C in tryptic soy broth containing 20% glycerol.

### Biofilm Preparation

The mixed-species biofilm model is based on a batch culture approach using saliva-coated hydroxyapatite (sHA) discs, and was designed to mimic the formation of biofilms according to “ecological plaque-biofilm” concept [17] as described by Koo et al. [12] and Xiao et al. [13] (details of this model are depicted in the Figure S1A). *Streptococcus mutans* UA159, *A. naeslundii* ATCC 12104 and *S. oralis* ATCC 35037 cells were grown in ultra filtered (10 kDa molecular-weight cut-off membrane; Prep/Scale, Millipore, MA) buffered tryptone-yeast extract broth (UFTYE; 2.5% tryptone and 1.5% yeast extract, pH 7.0) with 1% glucose at 37°C and 5% CO_2_ to late-exponential phase (OD_600 nm_ 1.0 for streptococci and OD_600 nm_ 1.5 for *A. naeslundii*). For mixed-species biofilms, the bacterial suspensions were mixed to provide an inoculum with a defined microbial population of *S. mutans* (10^2^ colony-forming unit - CFU/ml), *A. naeslundii* (10^6^ CFU/ml), and *S. oralis* (10^7^ CFU/ml). For single-species biofilms, only *S.* *mutans* (10^2^ CFU/ml) was added to the culture medium. Biofilms were formed on hydroxyapatite discs (1.25 cm diameter, Clarkson Chromatography Products, Inc., South Williamsport, PA) coated with filter-sterilized clarified human whole saliva (sHA), which were placed in a vertical position using a custom-made disc holder [12], [20]. The mixed population of *S. mutans*, *A. naeslundii* plus *S. oralis* and the single-species population (*S. mutans* only) were inoculated separately in 2.8 ml of UFTYE with 0.1% sucrose, and incubated at 37°C and 5% CO_2_. During the first 19 h, the organisms were grown undisturbed to allow initial biofilm formation. At 19 h, the culture medium was replaced by transferring the custom-made disc holder with biofilms using a forceps to wells with fresh media (UFTYE containing 0.1% sucrose). The biofilms were grown until 29 h for establishment of mixed-species community [12]. At 29 h of biofilm growth, both mixed-species and *S. mutans* biofilms were transferred to UFTYE containing 1% sucrose to induce environmental changes to simulate a cariogenic challenge. The culture medium was then changed twice daily (at 8 am and 6 pm) until the end of the experimental period. At 29 h, *S. oralis* (a non-cariogenic species) is the predominant species; but an ecological shift occurs after the introduction of 1% sucrose and *S. mutans* becomes the dominant species in this mixed-species system at 115 h. The dynamic changes in the proportions of *S. mutans, S. oralis* and *A. naeslundii* observed in our model [12], [13] are in line with those found in dental plaque linked with dental caries development (as reviewed by Marsh [1] and Takahashi and Nyvad [10]).

### *S. mutans* Proteome Analysis

#### Sample preparation for mass spectrometry

At 67 and 115 h of biofilm experiment, biofilms were removed, transferred to 2 ml of 0.89% NaCl solution containing protease cocktail inhibitor (Roche) and homogenized by two sonication steps. The first step was done by water bath sonication to help remove biofilms from the sHA surface (5 min/cold water). Then, the biofilms were scraped off the HA discs with a sterilized metal spatula, the discs were removed and a second sonication using a probe was performed (30 sec, 7 watts, samples on ice). Aliquots were taken for bacteria population assessment by qPCR [21], [22] (because protease inhibitor affected CFU assessment; data not shown), and for total protein quantification (by acid digestion followed by ninhydrin assay [23]). The remaining biofilms suspensions were centrifuged and the supernatants stored in 50 ml tubes. The cell pellets were washed twice with 0.89% NaCl solution containing protease cocktail inhibitor, and the supernatants were mixed with the first supernatant. The biofilm pellets (cellular fraction) and corresponding supernatants (soluble fraction) were frozen at −80°C, and next day the supernatant were lyophilized. Each cell pellet was suspended in 800 µl of lysis buffer (0.5% TritonX-100, 200 mM DTT, 50 mM Tris HCl, pH 8.0) and the suspension was sonicated on ice-cold water for 60 minutes. 267 µl of 100% trichloroacetic acid (TCA) was added to each sample to achieve a final TCA concentration of 25%. Samples were vortexed briefly, incubated on ice for 3 hours and spun at 14,000 rpm for 10 minutes. The supernatant was discarded. The pellet was washed twice with 200 µl of ice-cold acetone and acetone was discarded after spinning at 14,000 rpm for 5 minutes. Pellets and soluble fractions were treated with 150 µl of 100 mM Tris-HCl (pH 8.5) with 8 M urea, and agitated to dissolve proteins. 6 µL of 0.5 M DTT was added and the samples were shaken at 37°C for 15 minutes. Samples were cooled to room temperature (RT) and 12 µl of 0.25 M iodoacetamide was added. Samples were incubated for 20 minutes in the dark at RT. 600 µl of 100 mM Tris-HCl (pH 8.5) was added so that final urea concentration was <2 M. Trypsin was added in a 1∶100 ratio and samples were shaken in a bench-top shaker at 37°C overnight. Formic acid was added to a final concentration of 4% and samples were spun at 14,000 rpm for 20 minutes. Samples were transferred to another eppendorf tube and 100 µg total protein was removed for loading onto a microcapillary column.

#### MudPIT

Each protein digest was pressure-loaded onto a fused silica capillary column containing 2.5 cm of Partisphere strong cation exchanger (SCX) (Whatman, Clifton, NJ) followed by 2.5 cm of 10 µm Aqua C18 (Phenomenex, Ventura, CA) packed into a 250-µm i.d. capillary (Polymicro Technologies, Phoenix, AZ) with a 1 µm frit. The column was washed for 60 minutes with buffer containing 95% water, 5% acetonitrile, and 0.1% formic acid. After washing, a 100–µm i.d. capillary with a 5-µm pulled tip packed with 10 cm 3-µm Aqua C18 material (Phenomenex, Ventura, CA) was attached via a union. The entire split-column was placed in line with an Agilent 1100 quaternary HPLC (Palo Alto, CA) and analyzed using a modified 12-step separation similar to those described previously [14]. The buffer solutions used were 5% acetonitrile and 0.1% formic acid (buffer A), 80% acetonitrile and 0.1% formic acid (buffer B), and 500 mM ammonium acetate, 5% acetonitrile and 0.1% formic acid (buffer C). Step 1 consisted of a 60 min gradient: 0–15 minutes, 0–50% buffer B; 15–40 minutes, 50–90% buffer B; 40–60 minutes, 90-0% buffer B. Steps 2–12 had the following profile: 15 min of X% buffer C, a 45 min gradient from 0–50% buffer B, a 40 min gradient from 15–100% buffer B and a 20 minute gradient from 100-0% Buffer B. The 15 min buffer C percentages (X) were 5, 10, 20, 30, 40, 50, 60, 70, 80, 90 and 100%, respectively, during the 12-step analysis.

As peptides eluted from the microcapillary column, they were electrosprayed directly into an LTQ-Orbitrap mass spectrometer (ThermoFinnigan, Palo Alto, CA) with the application of a distal 2.4 kV spray voltage. A cycle of one full-scan mass spectrum (400–1400 m/z) followed by 8 data-dependent MS/MS spectra at a 35% normalized collision energy was repeated continuously throughout each step of the multidimensional separation. Application of mass spectrometer scan functions and HPLC solvent gradients were controlled by the Xcalibur datasystem.

#### Analysis of tandem mass spectra

The acquired MS/MS spectra were analyzed using the following software analysis protocol. MS/MS spectra were searched with the ProLuCID algorithm [24] or the Sequest algorithm [25] against the NCBI-RefSeq *S. mutans* database (01/01/2010) and an *S. oralis* database (01/01/2010) concatenated to a decoy database in which the sequence for each entry in the original database was reversed [26]. All searches were parallelized and performed on a Beowulf computer cluster consisting of 100 1.2 GHz Athlon CPUs [27]. Peptides within 3 amu mass tolerance of the precursor mass and with one or two tryptic ends were considered during the database searches. ProLuCID results were assembled and filtered using the DTASelect (version 2.0) program [28], [29]. DTASelect 2.0 uses a linear discriminant analysis to dynamically set XCorr and DeltaCN thresholds for the entire dataset to achieve a user-specified false positive rate (0.1% in this analysis). The false positive rates are estimated by the program from the number and quality of spectral matches to the decoy database. Therefore, the *S. mutans* proteins were detected and discriminated from *S. oralis* and *A. naeslundii* in the proteome analyses based on unique peptide sequences. Unique peptide sequences are sequence regions that are different between two closely related protein sequences [14], [15]. The use of uniquely identified peptides enables the discrimination between proteins from different species that are closely related, i.e. *S. mutans* and *S. oralis*.

We focused on proteins encoded by *S. mutans* UA159. MudPIT analysis was used to provide a profile of (i) the identity and (ii) abundance (based on spectral counts) of the proteins detected in each condition and time point. The software ProteinCenter was used to categorize the proteins detected in gene ontology (GO) of biological processes; and DAVID to verify the KEGG (Kyoto Encyclopedia of Genes and Genomes) pathways to which the proteins identified belonged to. For protein abundance, the spectrum count was determined by 100 µg of total protein (n = 2). The proteome data were normalized using the number of viable cells of *S. mutans* (based on CFU values) present in the biofilms collected at each time point and condition. In each experiment, extra biofilms were grown side by side to those biofilms used for proteome and processed for platting; the CFU values obtained were used for data normalization. We also confirmed the number of viable cells in the samples submitted to MudPIT by a secondary method based on qPCR and propidium monoazide (which allows amplification of genomic DNA from viable cells only [22]). The number of viable cells calculated by qPCR matched with that from CFU determination. We used CFU values because this was the method of choice during the establishment of our mixed-species biofilm model [12], [13]. Thus, the level of *S. mutans* protein abundance in mixed-species biofilms was calculated by normalizing the spectrum count to the relative numbers of viable *S. mutans,* i.e. multiplying the spectrum count values by the ratio of *S. mutans* CFU to the total CFU in the mixed-species samples.

### *S. mutans* Gene Expression Analysis using Reverse Transcription Quantitative PCR (RT-qPCR)

The biofilms were analyzed at 43, 67, 91, and 115 h of biofilm development. RNA was extracted and purified using standard protocols optimized for biofilms [30]. The RNA integrity number for all of our samples was ≥8.0, as determined by on-chip capillary electrophoresis with the Agilent 2100 Bioanalyzer (Agilent Technologies, Inc., Santa Clara, CA). We performed RT-qPCR to measure the expression profile of *S. mutans* specific genes directly associated with extracellular polysaccharide matrix development (*gtfB, gtfC, ftf, dexA, gbpB);* genes involved with ability to cope with acidic environments and other stresses (stress tolerance mechanisms*) (atpD, fabM, groES, nox);* and genes involved with sugar metabolism *(glgP* and *manL).* Briefly, cDNAs were synthesized using 0.5 µg of purified RNA and the BioRad iScript cDNA synthesis kit (Bio-Rad Laboratories, Inc., Hercules, CA). To check for DNA contamination, purified total RNA without reverse transcriptase served as a negative control. The resulting cDNA and negative controls were amplified by a Bio-Rad CFX96 system (Bio-Rad Laboratories, Inc., CA) using specific primers and TaqMan probes (Table S1) and iQ Multiplex Powermix for multiplex reactions or iQ Supermix for singleplex reactions. When Taqman probes were not avalilable, cDNAs and controls were amplified using iQ SYBR Green supermix (Bio-Rad Laboratories) and specific primers [31]. A standard curve was plotted for each primer set as detailed elsewhere [32]. The standard curves were used to transform the critical threshold cycle (Ct) values to the relative number of cDNA molecules. Relative expression was calculated by normalizing each gene of interest to the *S. mutans* 16S rRNA gene, which is a well-established reference gene [33], [34]. The qPCR runs of a specific gene or a set of specific genes were done side by side with 16S rRNA to reduce variability and enhance reproducibility. The normalization was performed for each experimental sample from 6 distinct experiments in duplicates (n = 12). All *S. mutans* specific primers were tested for cross reactivity to ensure that gene expression of *S. mutans* can be discriminated from the other species. None of the *S. mutans* primers cross reacted with either *S. oralis* or *A. naeslundii* (data not shown).

An exploratory data analysis of the RT-qPCR data was performed to determine the most appropriate statistical test; the assumptions of equality of variances and normal distribution of errors were also checked. The data were then analyzed using ANOVA, and the F test was used to test any difference between the groups (distinct time points within same experimental condition and different experimental conditions). When significant differences were detected, a pairwise comparison was made between all the groups using Tukey-Kramer HSD method to adjust for multiple comparisons. Statistical software JMP version 3.1 (SAS Institute, Cary, NC, USA) was used to perform the analyses. The level of significance was set at 5%.

## Results and Discussion

In this study, we used high-throughput, quantitative proteomics profiling to identify relevant proteins expressed by *S. mutans* during the development of mixed-species biofilms according to an ecological model. Our results show how *S. mutans* orchestrates the expression of gene products from distinct pathways (some that overlap) in response to sucrose that facilitates its establishment and optimal survival, in the presence of other bacteria, within a dynamically changing biofilm milieu over time.

### *S. mutans* Survival within Ecological Mixed-species Biofilm Model

Oral biofilms are comprised of mixed microbiota *in vivo*. The transition from non-pathogenic to pathogenic biofilm involves environmental changes (i.e. introduction of sucrose) that dramatically influence the microbial and the biochemical composition of biofilm. The mixed-species model used here was designed to mimic the ecological and the biochemical changes associated with cariogenic biofilm assembly [12], [13], which include: 1) microbial population shift between initially low abundance *S. mutans* and the early colonizers present in high numbers (e.g. *S. oralis*); 2) the introduction of sucrose as environmental challenge, causing biochemical changes (EPS and acid production) and microbial shifts towards dominance of *S. mutans* at later stages of biofilm formation; and 3) spatiotemporal changes of 3D architecture and environmental pH (Figure S1). We selected two specific time points for proteomic analysis that are based on the population shifts, EPS-matrix and microcolony assembly observed in our mixed-species biofilm model. We selected 67 h because at this time point *S. mutans* start to shift from initially very low numbers to a major co-habitant while at 115 h *S. mutans* become the dominant species (Figure S1B). Furthermore, we also used single-species *S. mutans* biofilms to examine how *S. mutans* protein synthesis is affected by the presence of additional organisms.

### Overview of *S. mutans* Proteomic Responses during Biofilm Development

**Figure 1:**
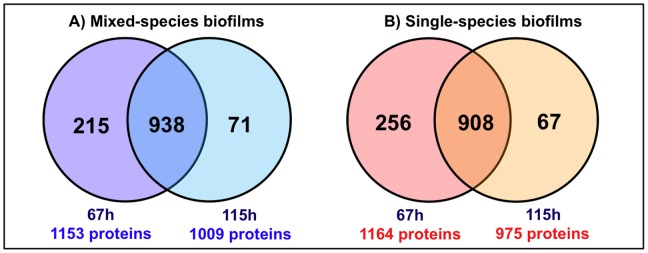
Number of *S. mutans* UA159 proteins detected in the biofilms. From 1966 proteins encoded by this organism. A) Mixed-species biofilms *(S. mutans, A. naeslundii* and *S. oralis*) and B) Single-species biofilms (*S. mutans* alone). Among the common proteins at 67 and 115 h, 817 were detected in both single and mixed-species biofilms. Number of proteins in mixed- U single-species at 67 h is 87 proteins, whereas mixed- U single-species at 115 h are 12 proteins. The number of proteins detected exclusively in mixed-species biofilms was 70 and 19 at 67 and 115 h, respectively; and in single-species biofilms was 51 and 25 at 67 and 115 h, respectively.

The MudPIT approach detected up to 60% of the proteins encoded by *S. mutans* in biofilm samples (Figure 1; Table S2), which is significantly higher output than standard proteomic analysis of *S. mutans* using 2D gel electrophoresis [35], [36], [37], [38], [39]. Figure 1 shows the number of *S. mutans* proteins detected per time point and type of biofilm evaluated.

**Figure 2:**
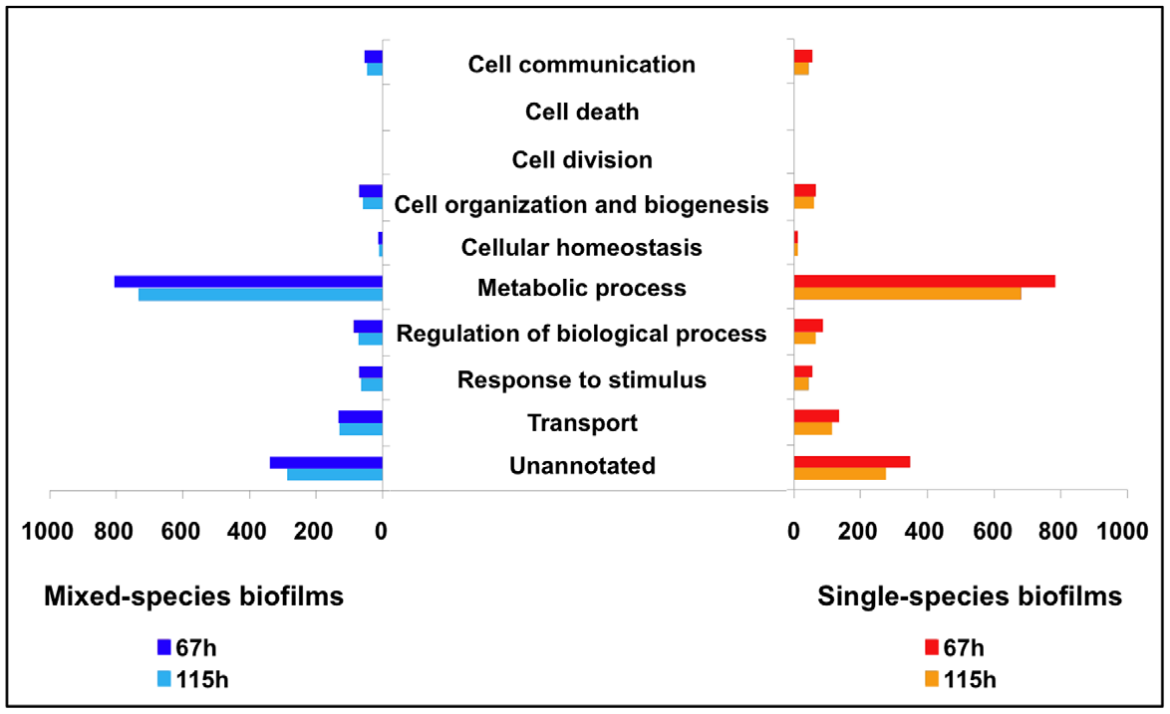
Distribution of *S. mutans* UA159 proteins into specific gene ontology (GO) of biological processes. The organization was performed via Proteome Center Software.

**Figure 3:**
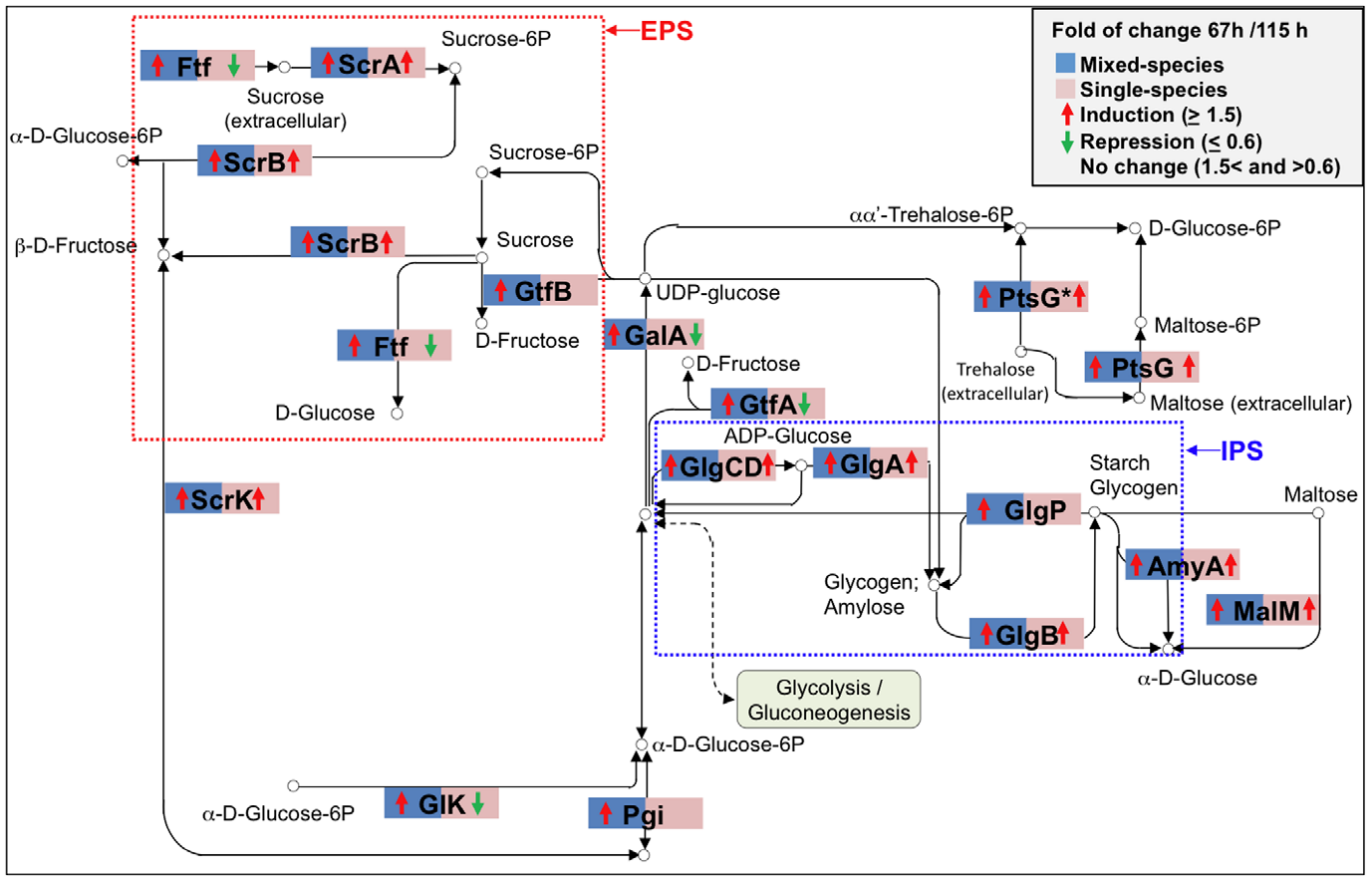
Sucrose and starch metabolism pathway. Adapted KEGG pathway with proteins detected by MudPIT analysis highlighted. The blue square represents mixed-species biofilms; and the red square represents *S. mutans* biofilms. The fold of change of protein synthesis from 67 to 115 h is indicated by arrows; fold ≥1.5 represents induction (red ↑); fold ≤0.6 represents repression (green ↓); and no arrows are depicted for fold 1.5< and >0.6. The proteins represented in this diagram are important for *S. mutans* biofilm formation and survival: sugar uptake (ScrA, ScrB, PtsG, MalM), EPS matrix (Ftf, GtfB), intracellular polysaccharide storage (AmyA, GlgA, GlgB, GlgP). * Plus TreA.

The distribution of proteins identified in gene ontology (GO) categories of specific biological processes showed that the highest number was detected for proteins involved in *S. mutans* metabolic process, followed by unannotated (i.e. uncharacterized) proteins (Figure 2). In addition, the analysis of KEGG (Kyoto Encyclopedia of Genes and Genomes) pathways showed that most proteins detected in both mixed- and single-species biofilms belong to (i) starch and sucrose metabolism (carbohydrate metabolism); (ii) purine metabolism (nucleotide metabolism), (iii) pyrimidine metabolism (nucleotide metabolism); and (iv) aminoacyl-tRNA biosynthesis (protein synthesis) metabolic pathways. As an example, we show the proteins differentially expressed in the starch and sucrose metabolism pathway (Figure 3), which contains some of the critical factors associated with EPS-matrix formation and carbohydrate metabolism.

Furthermore, the MudPIT analyses provided quantitative data of the production (i.e. abundance) of each *S. mutans* protein detected in the biofilm samples tested. The proteins Tuf (elongation factor Tu), Eno (phosphopyruvate hydratase), and DnaK (molecular chaperone) are among the most abundant proteins in all conditions evaluated (Table S2). The MudPIT coupled with spectrum counting is advantageous because it is a label-free large-scale and quantitative proteomic approach [14], [15]. It is a well-proven method for the identification and quantification of almost all proteins types from complex mixtures [14], [15], such as those found in biofilms despite the inherent limitations of the technique (i.e. spectrum counting is not an absolute quantification and may underestimate proteins that are present in very low amounts [15]).

We also analyzed the temporal effect and the presence of other species on *S. mutans* proteome data. In general, a higher number of proteins were detected at 67 h of biofilm formation for both mixed- and single-species communities (Figure 1). More importantly, the majority of the proteins in most biological processes (such as EPS synthesis and acid tolerance mechanisms) are detected in high abundance at 67 h of mixed-species biofilm development (Figure 2; Table S2). The data suggest that *S. mutans* cells are highly active with enhanced fitness at 67 h (when *S. mutans* is trying to outcompete the other species in our model) than at 115 h (when *S. mutans* is already dominant in the biofilm milieu). The proteome profile of *S. mutans* grown in a mixed-species community has many differences but also similarities with that from *S. mutans* forming biofilms alone over time. For example, *S. mutans* proteins involved with EPS metabolism and specific acid tolerance mechanisms are highly abundant in mixed-species biofilms at 67 h, while their profile does not change much over time in single-species biofilm. In contrast, the profile of the proteins associated with sugar uptake and intracellular polysaccharide storage metabolism are similar irrespective of whether *S. mutans* is forming biofilms alone or mixed with other bacterial species (Figure 3; Tables 1; 2; S1).

It is critical to keep in mind that sucrose has dual function in assembling virulent biofilms: energy generation resulting in acid production and EPS matrix formation. The acid produced by bacterial cells entrapped within the EPS-enmeshed microcolonies cannot be readily neutralized, creating various low pH microenvironments within the complex biofilm 3D architecture (Figure S1; [13]). Thus, the acidic niches create favorable conditions for acid-tolerant bacteria to prosper, ensuring continued localized acid production. To thrive in this highly organized, acidic environment and changing ecosystem, *S. mutans* has sophisticated mechanisms to cope with fluctuation of dietary nutrients and environmental stresses, such as pH [40]. Therefore, we focused on essential metabolic pathways that may be important for *S. mutans* fitness in a cariogenic model, including proteins related to: (i) EPS synthesis and remodeling (Table 1); (ii) intracellular polysaccharide storage (IPS) and lipoteichoic acid metabolism (Table 2); and (iii) acid tolerance response mechanisms (Tables 3 and 4).

**Table 1:** Proteins related to EPS synthesis and remodeling.

Gene name	Protein abundance (Spectral counting*)				Description
	Mixed-species 67 h	Mixed-species115 h	*S. mutans*67 h	*S. mutans*115 h	
*gtfB* **	604	106	190	159	glucosyltransferase-I
*gtfC* **	973	201	313	322	glucosyltransferase-Si
*gtfD* **	79	51	60	56	glucosyltransferase-S
*dexA* **	193	46	49	41	dextranase precursor
*fruA* **	120	32	29	15	fructan hydrolase; exo-beta-D-fructosidase; fructanase, FruA
*sacB ftf* **	215	124	117	218	levansucrase precursor; beta-D-fructosyltransferase
*gbpA*	261	167	179	162	glucan-binding protein A, GbpA
*gbpB*	1200	486	201	753	putative secreted antigen GbpB/SagA; putative peptidoglycan hydrolase
*gbpC*	63	39	32	41	glucan-binding protein C, GbpC
*gbpD*	17	0	6	5	putative glucan-binding protein D; BglB-like protein
*vicK*	18	4	6	3	putative histidine kinase CovS; VicK-like protein
*vicR*	77	29	60	24	putative response regulator CovR; VicR-like protein
*gcrR*	164	60	55	43	response regulator GcrR for glucan-binding protein C
*luxS*	18	8	7	12	S-ribosylhomocysteinase
*rpoA*	101	162	97	44	DNA-directed RNA polymerase subunit alpha
*ccpA*	112	69	75	41	catabolite control protein A, CcpA
*manL*	142	69	75	73	putative PTS system, mannose-specific component IIAB

The protein abundance is represented by spectral counting (n = 2).

* Normalized by the numbers of *S. mutans* detected in each biofilm.

** Data for mixed-species at both time points and single-species biofilms at 67 h were published by Xiao et al. [13].

**Table 2:** Proteins related to IPS and lipoteichoic acid (LTA) metabolism.

Gene name		Protein abundance (Spectral counting)*				Description
		Mixed-species 67 h	Mixed-species115 h	*S. mutans*67 h	*S. mutans* 115 h	
IPS	*phsG glgP*	72	20	40	20	glycogen phosphorylase
	*glgA*	2	1	23		glycogen synthase
	*glgB*	20	2	30	8	glycogen branching enzyme
	*glgC*	31	10	19	5	glucose-1-phosphate adenylyltransferase
	*glgD*	15	6	24	4	putative glycogen biosynthesis protein GlgD
	*glg glgP* (SMU.1564)	212	101	99	90	putative glycogen phosphorylase
	*amyA*	2		7	2	cytoplasmic alpha-amylase
	*pulA*	4	3	6	4	putative pullulanase
LTA	*dltA*	59	26	34	19	D-alanine–D-alanyl carrier protein ligase
	*dltB*			1		D-alanyl transfer
	*dltC*	6	3	2	5	D-alanine–poly(phosphoribitol) ligase subunit 2
	*dltD*	88	11	38	13	putative extramembranal protein, DltD protein

The protein abundance is represented by spectral counting (n  = 2).

* Normalized by the numbers of *S. mutans* detected in each biofilm.

### Proteins Related to EPS Synthesis and Remodeling

The profile of abundance of *S. mutans* proteins implicated in EPS matrix synthesis (GtfB, GtfC, GtfD, Ftf), remodelling (DexA, FruA), adhesion (GbpA, GbpB, GbpC and GbpD), and regulation (VicRS, GcrG, LuxS, RpoA, CcpA and ManL) are shown in Table 1. Among them, GtfB, GtfC, and GbpB are detected in elevated amounts particularly at 67 h. GtfB synthesizes mostly insoluble glucans (rich in α1,3-linked glucose) while GtfC forms a mixture of insoluble and soluble glucans (mostly α1,6-linked glucose) [4]. Secreted GtfC is primarily incorporated into the tooth-pellicle whereas GtfB preferably attaches to the bacterial surfaces [4]. The insoluble and rigid α1,3-linked glucans produced by these surface-adsorbed Gtfs are essential for the assembly and structural integrity of the matrix and microcolonies, as well as for the maintenance of acidic pH microenvironments within biofilms [13]. GtfB and GtfC are also recognized virulence factors associated with pathogenesis of dental caries *in vivo* [5], [6], [41].

We also observed that specific regulators of expression of these exoenzymes were detected in *S. mutans* at 67 h of biofilm development. The two component system VicRK and the LuxS-based signaling system were identified, which have been shown to directly regulate glucan synthesis by GtfB and GtfC [42], [43]. Furthermore, CcpA and ManL were detected in high levels at early stage of biofilm development; they are also implicated with up-regulation of EPS synthesis [44], [45], [46]). Altogether, these observations are congruent with the pattern of Gtfs detected in our biofilm system.

It is also noteworthy that the presence of dextranase (DexA) and fructosyltransferase (Ftf) (albeit less abundant than GtfB and GtfC) may have direct implications on the initial assembly of an insoluble EPS-matrix and with the acidification of the biofilm microenvironment. DexA digest soluble α1,6-linked glucans which provides (i) small dextrans which serve as acceptors for synthesis of insoluble glucans by GtfB, and (ii) provide additional substrates for acid production [47], [48], [49], [50]. Fructans formed by Ftf provide storage of extracellular nutrients, and have high water regain value that help to keep biofilm hydrated [51].

Conversely, synthesis of glucan binding proteins (Gbps) may enhance the ability of *S. mutans* to interact with the EPS-rich matrix [52]. The adhesion between the bacterial cells and the EPS-matrix may be mediated in part through cell-surface GbpC, and possibly GbpB whereas secreted GbpA and GbpD may be cross-linked with the matrix contributing to the maintenance of the biofilm architecture [53]. The elevated amounts of GbpB observed here may have direct implications for the biofilm morphogenesis and structural integrity, because a conditional mutant for this protein has impaired biofilm accumulation [54].

The presence of proteins associated with EPS synthesis in one hand with others involved with glucan binding processes illustrates how *S. mutans* builds up the biofilms after the introduction of sucrose, particularly at 67 h (a critical time point where the microbial population shifts occurs towards *S. mutans* dominance in our biofilm model). We conducted RT-qPCR analysis of selected genes at 67 and 115 h as well as the preceding time points of 43 and 91 h to examine the dynamics of gene expression associated with the proteins of interest. Clearly, the expression of *gtfB*, *gtfC* and *gbpB* were highly induced as the biofilm transits from 43 to 67 h (*P*<0.05) while their expression declines as *S. mutans* become the dominant species in the mature 115 h-biofilm (*P*<0.05), which agrees well with the quantitative proteome data. Although the fold of change in protein synthesis and gene expression does not present the same magnitude in all cases, the trend is conserved (either induction or repression over time).

Furthermore, genes related to EPS synthesis, remodeling and regulation are more highly expressed by *S. mutans* when grown in a mixed-species community that mimics the ecological plaque model than alone (*P*<0.05; Figure S2), confirming the proteomic profile between these two biofilm systems. Such differences could explain the structural disparity in the EPS-matrix and the size of microcolonies between mixed- and single-species biofilms (Figure S1).

Thus, the interplay of the gene products involved with glucan synthesis, degradation and binding provide an opportunity for *S. mutans,* which are initially in low number, to thrive in a mixed-species community by: 1) assembling EPS matrices on which the organism binds avidly through several Gbps, 2) providing additional carbohydrate sources for acid production, and 3) constructing highly cohesive bacterial islets (microcolonies) enmeshed in EPS, which facilitates the creation of acidic niches throughout the biofilm 3D architecture.

### Proteins Related to Intracellular Polysaccharide Storage (IPS) and Lipoteichoic Acid (LTA) Metabolism

**Figure 4:**
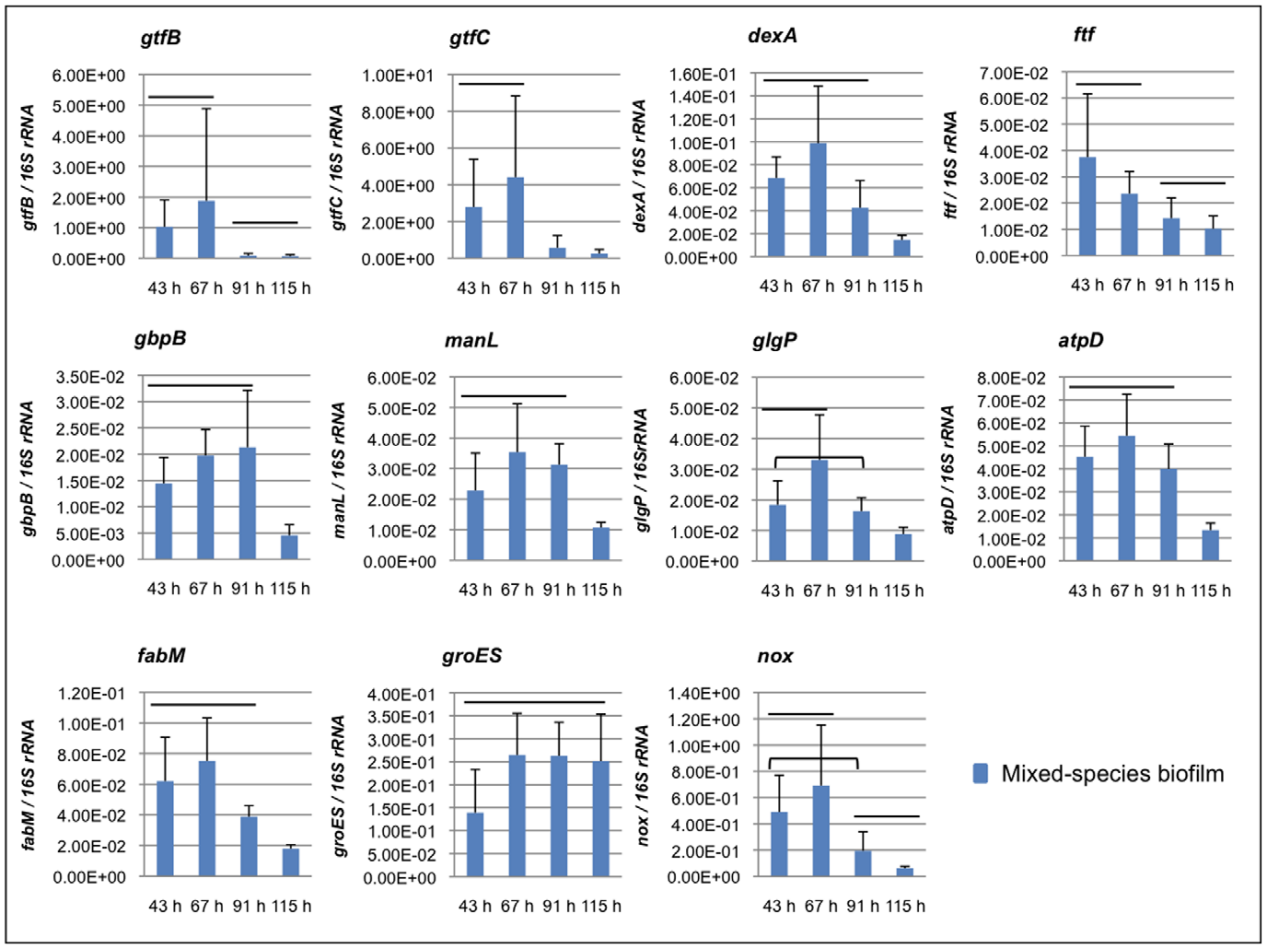
Dynamics of *S. mutans* gene expression during mixed-species biofilm development. Depicts selected *S. mutans* genes based on the proteome data. Comparison of gene expression data acquired at each time point for mixed-species biofilms (43 h vs. 67 h vs. 91 h vs.115 h) are shown; values connected by line are not significantly different from each other (*P*<0.05; n = 12).

IPS are glycogen-like storage polymers important for *S. mutans* virulence and are associated with the pathogenesis of dental caries [55], [56], [57]. IPS provide *S. mutans* with endogenous source of carbohydrates that can be metabolized when exogenous fermentable substrates have been depleted in the oral cavity. Proteins related to IPS metabolism are abundant at 67 h in mixed-species biofilms (Table 2). In particular, glycogen phosphorylase, a key enzyme in IPS metabolism/synthesis (see Figure 3), is detected in high levels at the earlier time point. The expression of gene *glgP* is significantly higher at 67 h than at the later time points (*P*<0.05, Figure 4), which agrees with the temporal trend seen in the proteome data for mixed-species biofilms. These findings indicate increased storage of IPS by *S. mutans* after introduction of sucrose as the biofilm start to accumulate. A similar profile of protein abundance and gene expression changes between 67 and 115 h was observed with single-species biofilms (Table 2, Figure S2), although some differences in the type of proteins were detected (e.g. high levels of glycogen synthase in single-species vs. high levels of glycogen phosphorylase in mixed-species).

We also observed another factor that may contribute with IPS accumulation by *S. mutans*. Proteins DltA, DltC, and DltD, involved with metabolism of LTA, were more abundant at 67 h, and were particularly elevated in mixed-species biofilms (Table 2). This observation is relevant because disruption of expression of *dltABCD* induced the synthesis of IPS [57], [58]. Whether these proteomic changes can actually increase the amounts of stored IPS in *S. mutans* within biofilms and how they are triggered by the presence of other organisms awaits further investigation. Furthermore, D-alanyl-LTA is involved with bacterial adhesion to hydroxyapatite and artificial surfaces, and initial biofilm formation process, possibly by incorporating LTA into the extracellular matrix [59], [60], [61]. Interestingly, the defective expression of *dltABCD* reduced acid tolerance of *S. mutans* grown in planktonic cultures [62].

Overall, the detection of proteins associated with IPS and LTA metabolism, which are particularly elevated at 67 h in mixed-species system provide additional insights on how S. mutans could establish themselves, survive and respond to an increasingly acidic and EPS-rich microenvironment following the introduction of sucrose.

### Proteins Related to Acid Stress Tolerance Response Mechanisms

The assembly of an insoluble EPS matrix and its spatial arrangement with bacterial cells creates acidic and protective microenvironments inside the microcolonies [13]. *S. mutans* have several mechanisms to cope with stressors such as low external pH and acidification of cytoplasm [2], [40]. Our data showed that *S. mutans* mounts an intricate yet interconnected response to adapt and to survive acidic stress, which are influenced by the presence of other organisms and biofilm age.

All proteins that encode the F1F0-ATPase system [63] for proton extrusion and ATP generation were detected in mixed-species biofilms (Table 3). Among them, AtpD was the most abundant protein, which has a critical function in the assembly of ATPase complex and is highly induced at low pH [64]. This complex helps to maintain the ΔpH across the bacterial membrane by pumping protons out of the cell. The temporal expression of gene *atpD* showed that from 43 to 67 h the expression is similar, and then significantly declines at 115 h (*P*<0.05), confirming the quantitative proteome data (Figure 4).

**Table 3:** Proteins related to acid and stress tolerance response: F1F0-ATPase and fatty acid metabolism (linked to membrane composition).

Gene name		Protein abundance (Spectral counting)*				Description
		Mixed-species 67 h	Mixed-species115 h	*S. mutans*67 h	*S. mutans*115 h	
F1F0-ATPase	*atpC*	22	28	16	20	F0F1 ATP synthase subunit epsilon
	*atpD*	357	245	157	205	F0F1 ATP synthase subunit beta
	*atpG*	182	20	62	17	F0F1 ATP synthase subunit gamma
	*atpA*	103	64	35	19	F0F1 ATP synthase subunit alpha
	*atpH*	9	8	6	19	F0F1 ATP synthase subunit delta
	*atpF*	48	14	11	10	F0F1 ATP synthase subunit B
fatty acid metabolism	*fabZ*	26	7	29	4	(3R)-hydroxymyristoyl-ACP dehydratase
	*bccP*	81	35	25	19	acetyl-CoA carboxylase biotin carboxyl carrier protein subunit
	*fabF*	228	98	118	57	3-oxoacyl-(acyl carrier protein) synthase II
	*fabG*	79	64	22	57	3-ketoacyl-(acyl-carrier-protein) reductase
	*fabD*	26	13	8	6	acyl-carrier-protein S-malonyltransferase
	*fabK*	195	146	209	78	putative trans-2-enoyl-ACP reductase
	*acpP*	72	47	25	62	acyl carrier protein
	*fabH*	4	2	2	4	3-oxoacyl-(acyl carrier protein) synthase III
	SMU.1745	4	5	3	3	putative transcriptional regulator
	*fabM*	234	4	249	8	enoyl-CoA hydratase
	SMU.1747	9		9	3	putative phosphatase

The protein abundance is represented by spectral counting (n  = 2).

* Normalized by the numbers of *S. mutans* detected in each biofilm.

Low pH triggers changes in the membrane fatty acid composition and also affects the permeability of the membrane to protons [65], [66]. All proteins encoded by the fatty acid biosynthetic gene cluster were also detected (Table 3). This cluster may be connected with ATPase system because the fatty acid composition is important for the optimal function of ATPpase, which is anchored to the membrane. In particular, FabM was detected in high levels at 67 h of mixed-species biofilm development (Table 3). FabM is responsible for the synthesis of monounsaturated fatty acids and is critical for *S. mutans* survival at low pH [65], [66]. The expression profile of gene *fabM* confirmed the trend of the protein detection between 67 and 115 h (Figure 4). Thus, the data suggest that *S. mutans* modulates specific changes in fatty acid profile in the membrane and the assembly of F1F0-ATPase system to ensure an optimal condition to control the protons level in the cytoplasm (and as a result the intracellular pH).

In addition, the proteins directly responsible for cytoplasm alkalinization are also detected in elevated amounts at 67 h of biofilm development (Table 4), which include: 1) the metabolism of branched chain amino acids (BCAA) [40], [67], 2) the malolatic fermentation (MLF) system [68], and 3) agmatine diamenase system (AgDS) (which also produces ATP that can be used for growth or to extrude protons via F1F0-ATPase system [69], [70]). Among them, metabolism of BCAA may have a significant role as its components are abundant (particularly IlvC), and they may have a synergistic role with F1F0-ATPase system and fatty acid composition in the membrane to enhance *S. mutans* survival in a low pH environment within biofilms. This may occur because *S. mutans* senses the low pH and modulate the carbon flux from acid production to BCAA biosynthesis [40], [67]. The abundance of MLF related proteins is rather low, and only one protein from the AgDS was detected in our analyses (Table 4), which is not surprising because *S. mutans* express AgDS at relatively low levels [69], [70]. Thus, the MLF and AgDS systems may have comparatively minor roles in *S. mutans* tolerance to acidic environment in the biofilms tested.

**Table 4:** Proteins related to acid and stress tolerance response: Proteins responsible for regulation of intracellular pH - branched chain amino acids (BCAA), malolatic fermentation (MLF), and agmatine diamenase system (AgDS).

Gene name			Protein abundance (Spectral counting)*				Description
			Mixed-species 67 h	Mixed-species 115 h	*S. mutans* 67 h	*S. mutans* 115 h	
BCAA		*ilvA*	50	10	40	11	threonine dehydratase
		*ilvB*	105	50	46	58	acetolactate synthase catalytic subunit
		*ilvC*	1207	603	1172	327	ketol-acid reductoisomerase
		*ilvE*	166	119	70	75	branched-chain amino acid aminotransferase
		*ilvH*	22	11	10	3	acetolactate synthase 3 regulatory subunit
		*livF*	39	17	33	7	putative branched chain amino acid ABC transporter, ATP-binding protein
		*livG*	64	14	36	12	putative branched chain amino acid ABC transporter, ATP-binding protein [Streptococcus mutans UA159]
		*livH*	0	2	0	0	putative branched chain amino acid ABC transporter, permease protein
		*livK*	383	117	246	125	putative ABC transporter, branched chain amino acid-binding protein
MLF	*mleR*		22	1	4	5	putative transcriptional regulator
	*mleS*		85	24	54	35	malate dehydrogenase
	*mleP*		0	0	0	0	
AgDS	*argR*		17	5	25	2	putative transcriptional regulator of arginine metabolism

The protein abundance is represented by spectral counting (n  = 2).

* Normalized by the numbers of *S. mutans* detected in each biofilm.

In summary, the F1F0-ATPase system, the membrane fatty acid biosynthesis and the BCAA metabolism appear to play major roles on acid tolerance, particularly when *S. mutans* is shifting from a minor to become a major resident within an increasingly acidic milieu found in the interior of microcolonies of mixed-species biofilm. However, the other mechanisms for acid stress adaptation (i.e. MLF and AgDS), even if having a minor role (based on protein abundance), may be also important for overall *S. mutans* fitness by helping to increase the cytoplasmic pH and generate ATP. The ATP generated can be used by F1F0-ATPase system to extrude protons from the cytoplasm. The expression of such interconnected mechanism is particularly important because the loss of one or more of the stress adaptive mechanisms can lead to a substantial reduction in *S. mutans* pathogenicity [2], [71].

### Other Proteins Related to Responses to Acid Stress

The exposure to acidic environment and other insults found in the biofilms may lead to accumulation of abnormal proteins. *S. mutans* (and other organisms) uses molecular chaperones and proteases to modulate the stability of proteins and prevent the accumulation of abnormal proteins by overseeing the correct folding [37], [72], [73]. The synthesis of chaperones GroEL, GrpE, DnaJ, DnaK and HtpX was elevated at 67 h of mixed-species biofilm development (Table S3). This finding makes sense because in our analyses the majority of the proteins are highly abundant at this time point, when *S. mutans* start to become a major co-habitant in the mixed-species biofilm. Therefore, the augmented production of chaperones ensures the quality of proteins being expresses, enabling *S. mutans* to thrive in this biofilm.

Although we recognize the importance of oxidative and osmotic stresses in the *S. mutans* physiology in biofilms, we did not analyze the data in greater detail to keep the focus on the acid stress processes. Nevertheless, we did detect high levels of NADH oxidase Nox (a major contributor to oxidative stress response [74]) and transcriptional repressor Rex (linked to coping with oxidative stress [75]), which may indicate that the access of oxygen to the *S. mutans* cells within the biofilms may be limited likely due to increase of thickness of the biofilms following introduction of sucrose.

In general, the profile of proteins and expression of genes associated with acid tolerance responses (and to other stresses) were different between mixed- and single-species biofilms (Tables 3 and 4; Figure S2). Most of the proteins are detected in elevated levels in mixed-species biofilms. The expression of selected *S. mutans* genes (*atpD, fabM, groES, nox*) was significantly higher in mixed-species biofilms (vs. single-species) at all time points (*P*<0.05; Figure S2). These differences are congruent with the overall observations between these two biofilm systems. Clearly, *S. mutans* growing in mixed-species biofilms has a distinctive fitness, allowing the bacterium to out-compete other co-habitants and to optimally survive the acidic milieu.

In addition, several uncharacterized proteins detected in this study could have an important role in *S. mutans* fitness and tolerance to environmental stresses within cariogenic biofilms (Table S2). Our data provide opportunities to investigate the function of these proteins in the expression of virulence by this pathogen, especially in the context of ecological biofilm concept. For example proteins encoded by genes SMU.1760 to SMU.1763, SMU.1337, SMU.210 are promising candidates for future studies [76]. The genes SMU.1760 to SMU.1763 are organized in o operon-like gene cluster, and their encoded proteins may be involved in stress response. SMU.1760, SMU.1761, SMU.1762 and SMU.1763 genes are all up-regulated in *S. mutans* lacking functional SpxA and SpxB [77], which have a global regulatory role in *S. mutans* stress response to acidic and oxidative environment. The proteins encoded by SMU.1337 (alpha/beta superfamily hydrolases with unassigned function) and SMU.210 (hypothetical protein with unknown function) are particularly abundant at 67 h (during microbial shifts favoring *S. mutans*) and 115 h (when *S. mutans* is the dominant species) of biofilm development, respectively. Each may have a distinctive role; SMU.1337 may be an important hydrolytic enzyme for *S. mutans* fitness and survival, while SMU.210 could be involved with persistence and stress adaptation. These proteins present homology to conserved hypothetical proteins from other bacteria (e.g. *Streptococcus pyogenes*, *Streptococcus gallolyticus* subsp. *gallolyticus*; *Streptococcus anginosus*; *Streptococcus downei*), and could have biological relevance to some pathogenic *Streptococcus* strains, deserving future investigation with defective mutant strains to pinpoint their exact role [76].

### Conclusions

The proteome analysis using multidimensional protein identification technology (MudPIT) revealed how *S. mutans* optimizes its metabolism and adapts, while enhancing its virulence and competitiveness, in response to a dynamically changing environment induced by sucrose within mixed-species biofilms. Moreover, the proteome data matched very well with the results from gene expression analyses using RT-qPCR, demonstrating the usefulness of this label-free quantitative proteomics approach to study the pathophysiological stage of microorganisms within complex biofilms over time.

Our study showed a complex interplay between gene products involved with EPS matrix assembly, remodeling and binding in one hand with specific processes associated with acid stress tolerance mechanisms, which are particularly induced when *S. mutans* is trying to outcompete other organisms (e.g. *S. oralis*) present in the biofilm system. In a simplified manner, the augmented production of EPS synthesis/remodeling and glucan-binding proteins helps to assemble a highly insoluble matrix that are uniquely arranged with bacterial cells forming microcolony complexes. At the same time, up-regulation of F1F0-ATPase system (e.g. AtpD), membrane fatty acids byosynthesis (e.g. FabM), and BCAA (e.g. IlvC) overlapping with molecular chaperones appears to be major responses by *S. mutans* (based on protein abundance and gene expression) to survive and adapt inside the microcolonies, which are highly acidic at 67 h of biofilm development in our system. These biological processes may be the major driving forces behind *S. mutans* successful establishment in mixed-species biofilms. Thus, novel therapies to control biofilm virulence expression should target them as a whole rather than a single pathway.

Clearly, the spatiotemporal regulation of this intricate yet interconnected network of pathways is highly complex and dynamic, and deserves further investigation both *in vitro* and *in vivo*.

## Supporting Information

Figure S1 Overall characteristics of the ecological mixed-species biofilm model. A) Experimental design; B) bacterial populational shift over time; C) representative 3D rendering images of mixed-species biofilm 115 h-old (green: bacteria; red: EPS), and a representative area showing microenvironmental pH within biofilm EPS-microcolony complex; and D) 3D structure of *S. mutans* single-species 115 h-old (adapted from Koo et al. [12]; Xiao et al. [13]).
(TIFF)

Figure S2 Comparison of *S. mutans* gene expression in mixed-species versus single-species biofilm at each developmental phase (*P<0.05).
(TIFF)

Table S1 Primers and TaqMan probes used for RT-qPCR.
(DOC)

Table S2 Proteome data generated by MudPIT.
(XLS)

Table S3 Proteins related to oxidative and osmotic stresses, and chaperones.
(DOC)
