# Supplementary figures and images for: Streptococcus mutans Protein Synthesis during Mixed-Species Biofilm Development by High-Throughput Quantitative Proteomics
Source: PLoS One. 2012 Sep 25;7(9):e45795. doi: 10.1371/journal.pone.0045795 (PMC3458072; doi:10.1371/journal.pone.0045795)

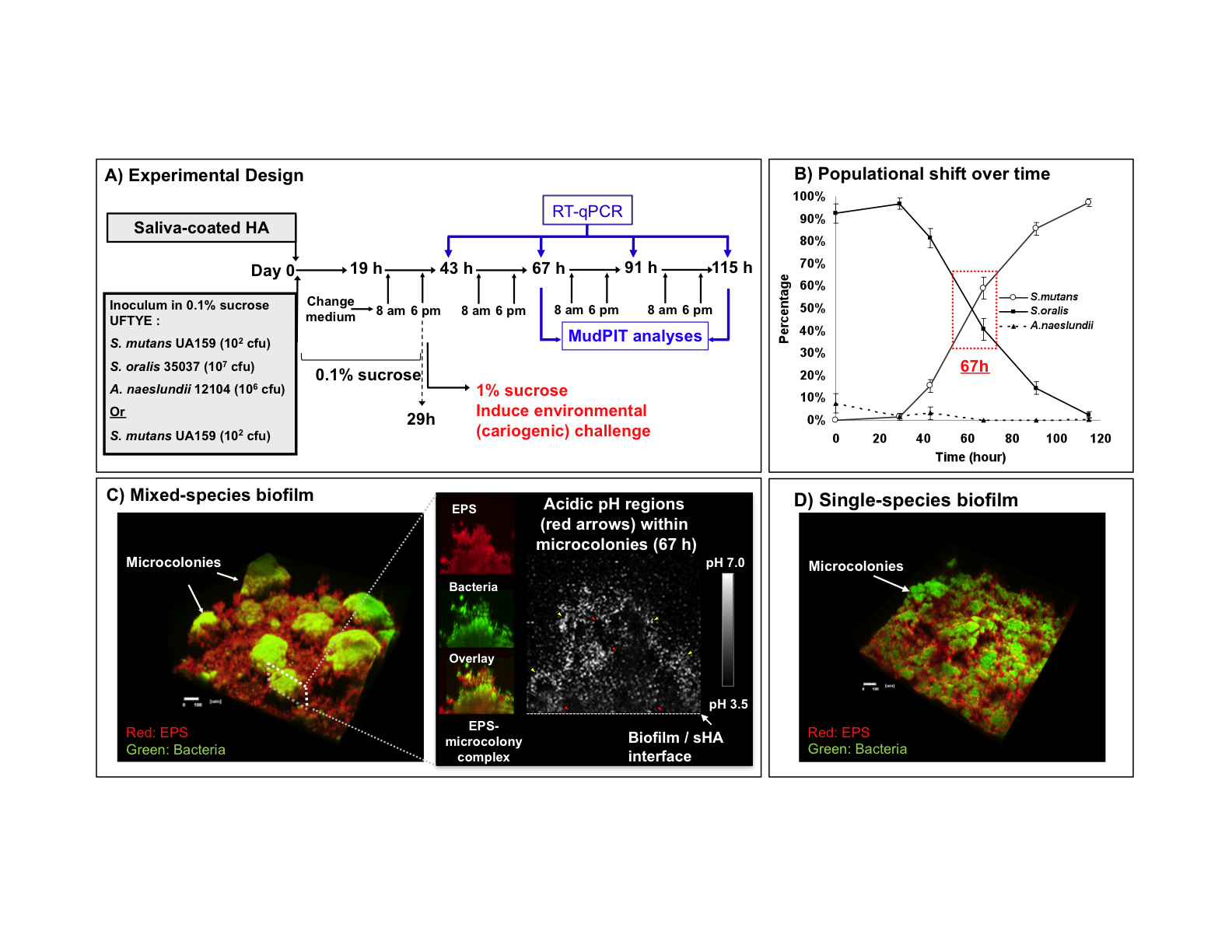

Supplement: Figure S1 — Overall characteristics of the ecological mixed-species biofilm model. A) Experimental design; B) bacterial populational shift over time; C) representative 3D rendering images of mixed-species biofilm 115 h-old (green: bacteria; red: EPS), and a representative area showing microenvironmental pH within biofilm EPS-microcolony complex; and D) 3D structure of S. mutans single-species 115 h-old (adapted from Koo et al. [12]; Xiao et al. [13]). (TIFF) [file pone.0045795.s001.tif]

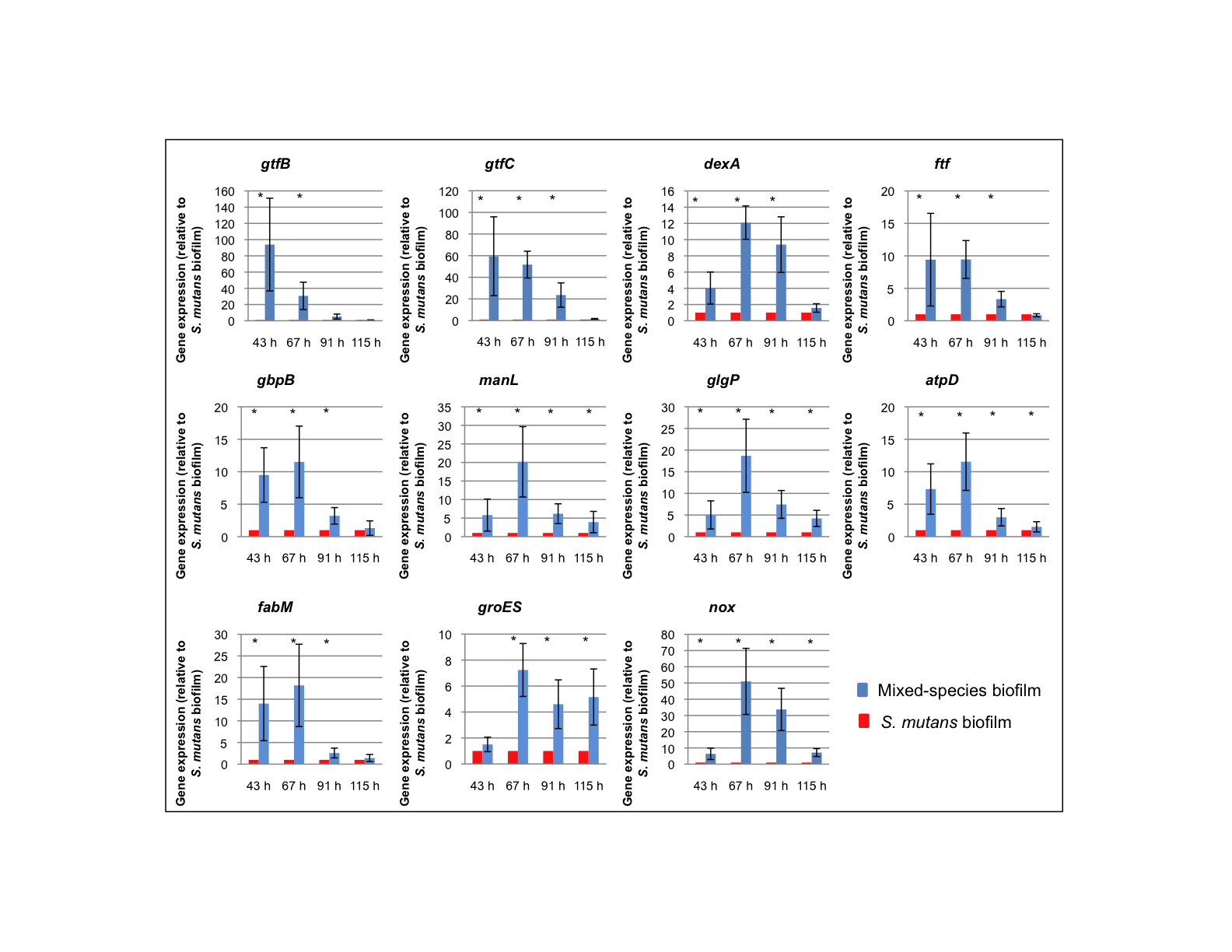

Supplement: Figure S2 — Comparison of S. mutans gene expression in mixed-species versus single-species biofilm at each developmental phase (*P<0.05). (TIFF) [file pone.0045795.s002.tif]
