# Supplementary material for: Streptococcus mutans Protein Synthesis during Mixed-Species Biofilm Development by High-Throughput Quantitative Proteomics
Source: PLoS One. 2012 Sep 25;7(9):e45795. doi: 10.1371/journal.pone.0045795 (PMC3458072; doi:10.1371/journal.pone.0045795)
Supplement: Table S1 — Primers and TaqMan probes used for RT-qPCR. (DOC) [file pone.0045795.s003.doc]

**Table S1**. **Primers and TaqMan probes used for RT-qPCR.**

| **GenBank Locus Tag** | **Gene Name** | **Primer sequence (forward and reverse)** | **TaqMan probe sequence and dual-labeled probes (reporters and quenchers)** | **Reference** |
| --- | --- | --- | --- | --- |
|  | *16S rRNA* | ACCAGAAAGGGACGGCTAAC | CTAACGCAATAAGCACTCCGCCTGG | [13] |
|  |  | TAGCCTTTTACTCCAGACTTTCCTG | 5’ FAM / 3’ BHQ-1 |
| SMU.1004 | *gtfB* | AAACAACCGAAGCTGATAC | ATTGGCTGCATTGCTATCATCA |
|  |  | CAATTTCTTTTACATTGGGAAG | 5’ HEX / 3’ BHQ-1 |
| SMU.1005 | *gtfC* | CTCTGACTGCTACTGATACAAG | AGCAACATCTCAACCAACCGCC |
|  |  | CCGAAGTTGTTGTTGGTTTAAC | 5’ Cal Fluor Red 610 / 3’ BHQ-2 |
| SMU.2042c | *dexA* | TATTTTAGAGCAGGGCAATCG | ACGCCAGTCATCCTCAACCGCA |
|  |  | AACCTCCAATAGCAGCATAAC | 5’ Quasar 705 / 3’ BHQ-2 |
| SMU.2028c | *ftf* | CTGACATAACTACGCCAAAG | CGCAATCTTACGAGCCTGTTCTGTT |
|  |  | TGCTTAAATTAATACCAGCTTC | 5’ HEX / 3’ BHQ-1 |
| SMU.1528c | *atpD* | GGCGACAAGTCTCAAAGAATTG | AGTCCATCACCGAGTTCAAGGGCAA | This study |
|  |  | AACCATCAGTTGACTCCATAGC | 5’ Cal Fluor Red 610 / 3’ BHQ-2 |
| SMU.1746c | *fabM* | ACTGATTAATGCCAATGGGAAAGTC | TCTCAACCAGATCGCCACCAACTGA |
|  |  | TGCGAACAAGAGATTGTACATCATC | 5’ Quasar 670 / 3’ BHQ-2 |
| SMU.1535c | *glgP* | GACTTTAAAGACACTCTGCATGAAG | ACCTGAAGCAACACCGACGGAACT |
|  |  | ACGAACAACCTTAGCCAAAGAAG | 5’ HEX / 3’ BHQ-1 |
| SMU.1877 | *manL* | ATGGCTATCGGAATCGTTATCG | AGATCATCAGGTCCTTCACTTGGCA |
|  |  | CAATTGCATCGTTAAACTTGGC | 5’ FAM / 3’ BHQ-1 |
| SMU.22 | *gbpB* | ATACGATTCAAGGACAAGTAAG | NA |
|  |  | TGACCCAAAGTAGCAGAC |  |
| SMU.1955 | *groES* | GAAAGAAGAAAAGGAACAAAC | NA | [31] |
|  |  | CACCAACAGCTACTACTT |  |
| SMU.1053 | *rex* | TGGTCGTGCTCTCCTGAATTATC | NA | Feng et al., personal communication |
|  |  | AGGAATACCGTCACTTGTCGTT |  |
| SMU.765 | *nox* | GGACAAGAATCTGGTGTTGA | NA |
|  |  | CAATATCAGTCTCTACCTTAGGC |  |

NA: not available. The RT-qPCR reactions were run using MyiQ qPCR detection system with iQ SYBR Green supermix (Bio-Rad Laboratories, Inc., CA, USA) and specific primers.
