# Supplementary material for: Streptococcus mutans Protein Synthesis during Mixed-Species Biofilm Development by High-Throughput Quantitative Proteomics
Source: PLoS One. 2012 Sep 25;7(9):e45795. doi: 10.1371/journal.pone.0045795 (PMC3458072; doi:10.1371/journal.pone.0045795)
Supplement: Table S3 — Proteins related to oxidative and osmotic stresses, and chaperones. (DOC) [file pone.0045795.s005.doc]

**Table S3: Proteins related to oxidative and osmotic stresses, and chaperones.**

| **Gene name** | | **Protein abundance (Spectral counting*)** | | | | **Description** |
| --- | --- | --- | --- | --- | --- | --- |
|  |  | **Mixed-species 67 h** | **Mixed-species 115 h** | ***S. mutans* 67 h** | ***S. mutans* 115 h** |  |
| Chaperones | *groEL* | 3393 | 1808 | 1359 | 1452 | chaperonin GroEL |
|  | *groES* | 248 | 484 | 190 | 234 | co-chaperonin GroES |
|  | *grpE* | 77 | 49 | 22 | 33 | heat shock protein GrpE |
|  | *dnaJ* | 4328 | 1630 | 2430 | 1963 | chaperone protein DnaJ |
|  | *dnaK* | 206 | 78 | 223 | 47 | molecular chaperone DnaK |
|  | *htpX* | 29 | 5 | 18 | 13 | heat shock protein HtpX |
| Osmotic stress | *trkB* | 48 | 28 | 18 | 35 | putative potassium uptake system protein TrkB |
| Oxidative stress | SMU.765 (*nox*) | 61 | 35 | 91 | 31 | NADH oxidase/alkyl hydroperoxidase reductase peroxide-forming |
|  | SMU.1053 (*rex*) | 26 | 7 | 13 | 9 | redox-sensing transcriptional repressor Rex |

* Normalized by the numbers of *S. mutans* detected in each biofilm.

The protein expression level is represented by Spectral counting (n = 2).
